# Supplementary material for: Visualization and Quantification of the Extracellular Matrix in Prostate Cancer Using an Elastin Specific Molecular Probe
Source: Biology (Basel). 2021 Nov 22;10(11):1217. doi: 10.3390/biology10111217 (PMC8615039; doi:10.3390/biology10111217)
Supplement: Supplementary file 1 [file biology-10-01217-s001.zip › biology-1421730-supplementary.pdf]

biology

MDPI

Article

Supporting Information

Elastin specific contrast agent for MRI to characterize prostate cancer

Avan Kader [1,2], Julia Brangsch [1], Carolin Reimann [1], Jan O. Kaufmann [1,4,5], Dilyana B. Mangarova [1,3], Jana Moeckel [1], Lisa Adams [1], Jing Zhao [1], Jessica Saatz [6], Heike Traub [6], Rebecca Buchholz [7], Uwe Karst [7], Bernd Hamm [1], Marcus R. Makowski [1,8,9]

1 Charité – Universitätsmedizin Berlin, corporate member of Freie Universität Berlin, Humboldt-Universität zu Berlin, and Berlin Institute of Health, Charitéplatz 1, 10117 Berlin, Germany; julia.brangsch@charite.de (J.B.); carolin.reimann@charite.de (C.R.); jan-ole.kaufmann@charite.de (J.O.K.); dilyana.mangarova@charite.de (D.B.M.); jana.moeckel@charite.de (J.M.); lisa.adams@charite.de (L.C.A); jing.zhao@charite.de (J.Z.); bernd.hamm@charite.de (B.H.); marcus.makowski@tum.de (M.R.M)

2 Department of Biology, Chemistry and Pharmacy, Institute of Biology, Freie Universität Berlin, Königin-Luise-Str. 1-3, 14195 Berlin, Germany

* Department of Veterinary Medicine, Institute of Veterinary Pathology, Freie Universität Berlin, Robert-von-Ostertag-Str. 15, Building 12, 14163 Berlin, Germany

4 Bundesanstalt für Materialforschung und -prüfung (BAM), Division 1.5 Protein Analysis, Richard-Willstätter-Str. 11, 12489 Berlin, Germany

5 Humboldt-Universität zu Berlin, Department of Chemistry, Brook-Taylor-Str. 2, 12489 Berlin, Germany

6 Bundesanstalt für Materialforschung und -prüfung (BAM), Division 1.1 Inorganic Trace Analysis, Richard-Willstätter-Str. 11, 12489 Berlin, Germany; Jessica.saatz@bam.de (J.S.); heike.traub@bam.de (H.T.)

7 Institute of Inorganic and Analytical Chemistry, Westfälische Wilhelms-Universität Münster, Münster, Germany (rebecca.buchholz@uni-muenster.de) (R.B.); uk@uni-muenster.de (U.K.)

8 King's College London, School of Biomedical Engineering and Imaging Sciences, United Kingdom, St Thomas' Hospital Westminster Bridge Road, London SE1 7EH, United Kingdom

9 Technical University of Munich, Department of Diagnostic and Interventional Radiology, Ismaninger Str.22, 81675 Munich, Germany

* Correspondence: avan.kader@charite.de

2.10. Laser ablation-inductively coupled plasma-mass spectroscopy (LA-ICP-MS)

System 1

A LSX 213 G2+ laser system (CETAC Technologies, Omaha, NE, USA) with a two volume HelEx II cell connected *via* Tygon tubing to an ICPMS-2030 (Shimadzu, Kyoto, Japan) were used for the measurement of the 1000 mm³ PC3-tumor sample. The samples were scanned at points with a size of 30 µm at a speed of 90 µm/s and 800 mL/min He as the transport gas. Standards were used for the quantification of ^{158}Gd, which are based on a gelatine standard (10% w/w) and are set at different concentrations of Gd from 1 to 500 µg/g. Three more isotopes, ^{31}P, ^{57}Fe and ^{64}Zn were measured in addition to the Gd in collision gas mode with He as the collision gas and an integration time of 100 ms. The limit of detection and the limit of quantification calculated with the 3σ- and 10σ-criteria, were 33 ng/g and 110 ng/g Gd.

System 2

LA-ICP-MS analysis of the 500 mm² PC3-tumor sample was performed on a commercial LA system (NWR-213, ESI, Bozeman, MT, USA) equipped with a two-volume sample

chamber coupled to a sector field ICP-MS (Element XR, Thermo Fisher Scientific, Bremen, Germany).

Helium was used as carrier gas with 1000 mL/min transporting the sample aerosol to the sector field ICP-MS, which was using the following parameters: sample time 0.002 s^{-1}, samples per peak 100, segment duration per isotope 0.01 s^{-1}, mass window per isotope 5%, search window 0%, integration window 5%. The samples were scanned with a spot size of 80 μm, 50 μm line spacing and a scan speed of 100 μm/s. The isotopes ^{31}P, ^{34}S, ^{57}Fe, ^{65}Cu, ^{66}Zn, ^{153}Eu, ^{160}Gd were measured in addition to ^{158}Gd.

Matrix-matched agarose gel standards cast on glass slides were used for drift control and calibration.[1] These standards contain Gd concentrations between 26.6 pg mm^{-2} and 600.1 pg mm^{-2}. Intensities of six line scans per standard were averaged for calibration.

Figure 5*: Western Blot analyse*

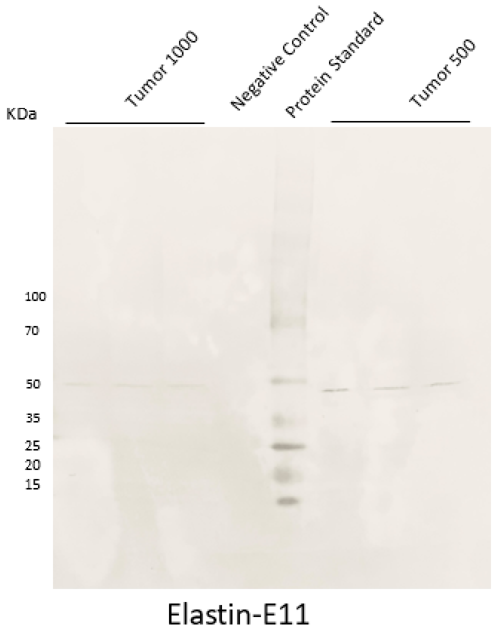

Figure 5.1*: Western Blot analyse for Elastin-E11.* *For each group, 3 tumors (n = 3 per group) were used for western blot analysis to detect the expression of elastin E-11. Intensity signal: Tumor 1000: left line 10.252, middle line 10.212 and right line 10.259; Tumor 500: left line 29.392, middle line 22.351 and 17.535.*

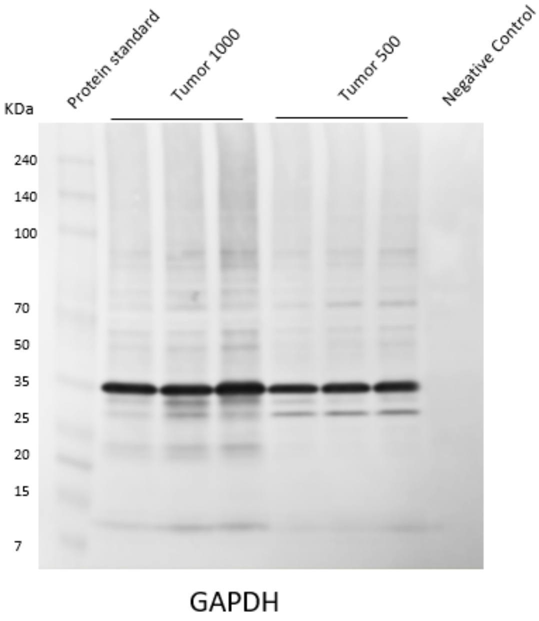

Figure 5.2*: Western Blot analyse for GAPDH.* *For each group, 3 tumors (n = 3 per group) were used for western blot analysis to control protein levels. Intensity signal: Tumor 1000: left line 18.874, middle line 17.081 and right line 17.516; Tumor 500: left line 15.588, middle line 15.394 and 15.547.*

1. Stärk, H.-J.; Wennrich, R. A new approach for calibration of laser ablation inductively coupled plasma mass spectrometry using thin layers of spiked agarose gels as references. *Anal. Bioanal. Chem.* **2011**, *399*, 2.
