# Supplementary figures and images for: Visualization and Quantification of the Extracellular Matrix in Prostate Cancer Using an Elastin Specific Molecular Probe
Source: Biology (Basel). 2021 Nov 22;10(11):1217. doi: 10.3390/biology10111217 (PMC8615039; doi:10.3390/biology10111217)

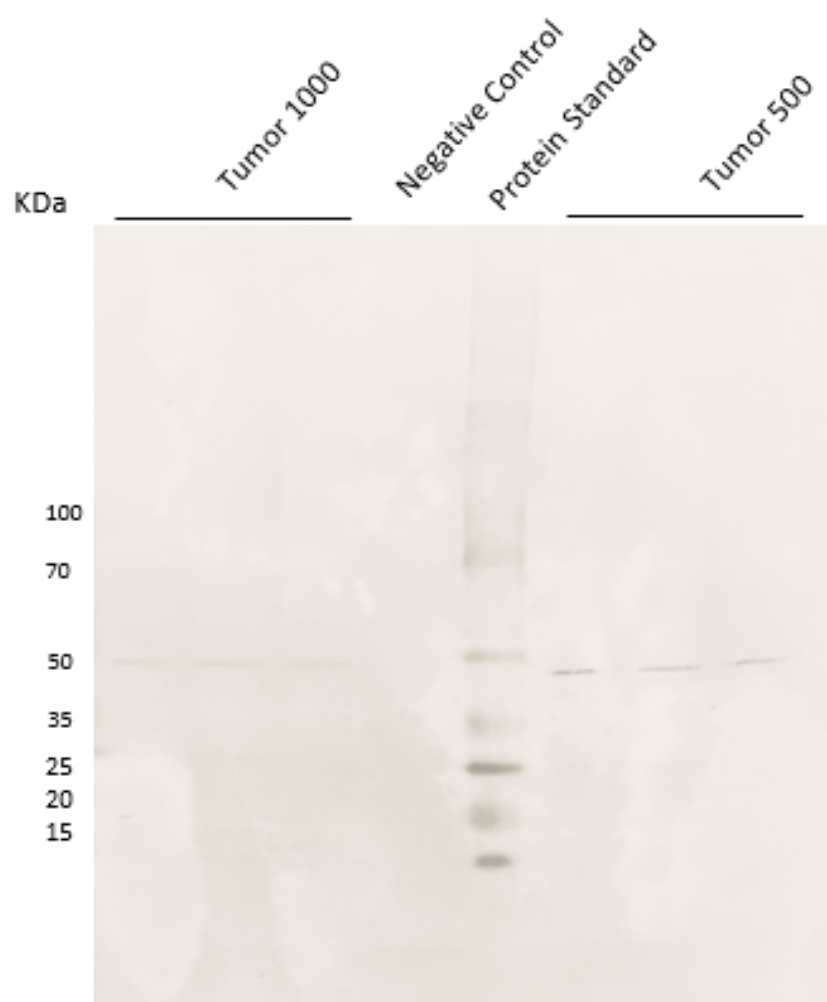

Elastin-E11

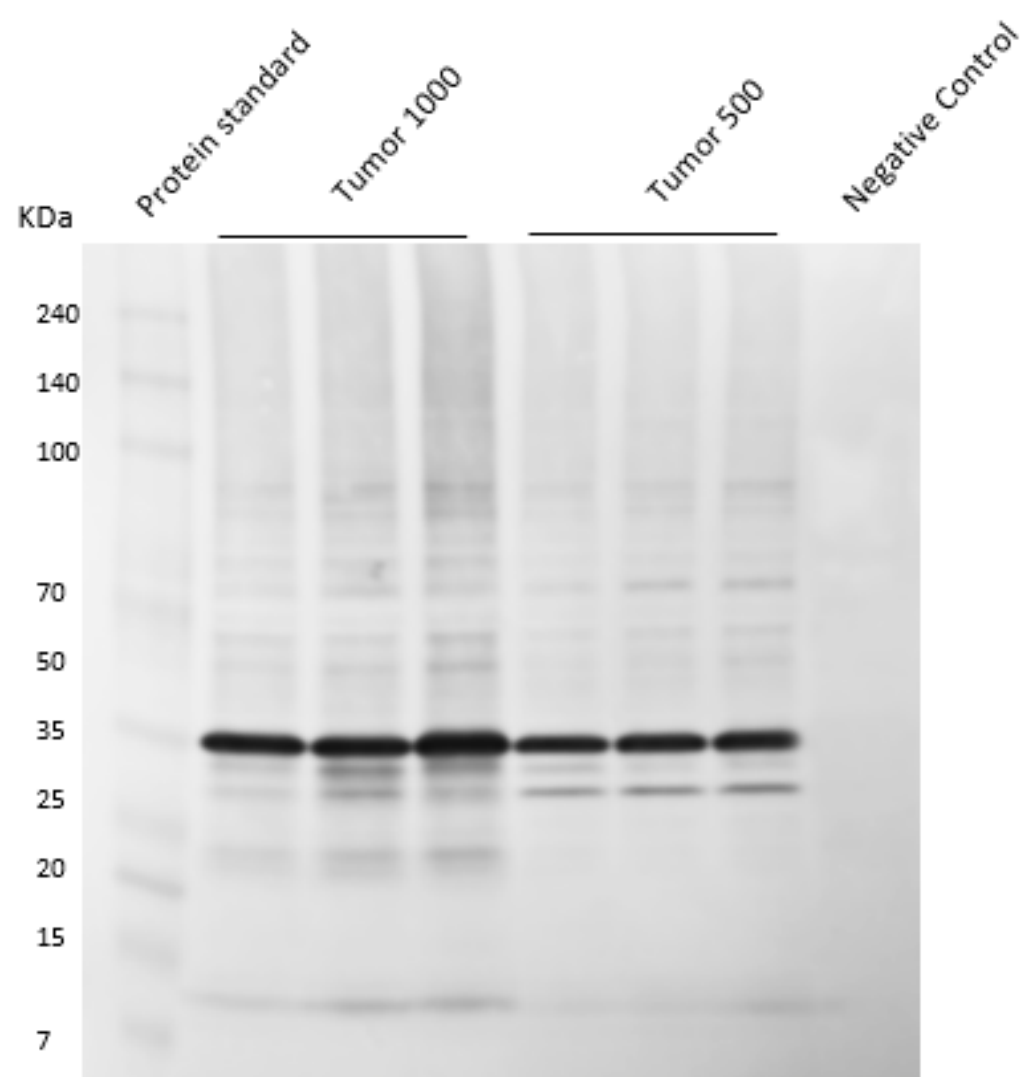

GAPDH

Supplement: Supplementary file 1 [file biology-10-01217-s001.zip › biology-1421730-original-images.pdf]
